# No Arachnoid Granulations—No Problems: Number, Size, and Distribution of Arachnoid Granulations From Birth to 80 Years of Age

**DOI:** 10.3389/fnagi.2021.698865

**Published:** 2021-07-01

**Authors:** Milan Radoš, Matea Živko, Ante Periša, Darko Orešković, Marijan Klarica

**Affiliations:** ^1^Croatian Institute for Brain Research, University of Zagreb School of Medicine, Zagreb, Croatia; ^2^Department of Molecular Biology, Ruđer Bošković Institute, Zagreb, Croatia; ^3^Department of Pharmacology, University of Zagreb School of Medicine, Zagreb, Croatia

**Keywords:** arachnoid granulations, cerebrospinal fluid, dural sinuses, CSF resorption, the classical concept of CSF physiology, the new concept of CSF physiology

## Abstract

**Introduction:** The study aims to quantify changes in the number, size, and distribution of arachnoid granulations during the human lifespan to elucidate their role in cerebrospinal fluid physiology.

**Material and Methods:** 3T magnetic resonance imaging of the brain was performed in 120 subjects of different ages (neonate, 2 years, 10 years, 20 years, 40 years, 60 years, and 80 years) all with the normal findings of the cerebrospinal fluid system (CSF). At each age, 10 male and 10 female subjects were analyzed. Group scanned at neonatal age was re-scanned at the age of two, while all other groups were scanned once. Arachnoid granulations were analyzed on T2 coronal and axial sections. Each arachnoid granulation was described concerning size and position relative to the superior sagittal, transverse, and sigmoid sinuses and surrounding cranial bones.

**Results:** Our study shows that 85% of neonates and 2-year-old children do not have visible arachnoid granulations in the dural sinuses and cranial bones on magnetic resonance imaging. With age, the percentage of patients with arachnoid granulations in the superior sagittal sinus increases significantly, but there is no increase in the sigmoid and transverse sinuses. However, numerous individuals in different age groups do not have arachnoid granulations in dural sinuses. Arachnoid granulations in the cranial bones are found only around the superior sagittal sinus, for the first time at the age of 10, and over time their number increases significantly. From the age of 60 onwards, arachnoid granulations were more numerous in the cranial bones than in the dural sinuses.

**Conclusion:** The results show that the number, size, and distribution of arachnoid granulations in the superior sagittal sinus and surrounding cranial bones change significantly over a lifetime. However, numerous individuals with a completely normal CSF system do not have arachnoid granulations in the dural sinuses, which calls into question their role in CSF physiology. It can be assumed that arachnoid granulations do not play an essential role in CSF absorption as it is generally accepted. Therefore, the lack of arachnoid granulations does not appear to cause problems in intracranial fluid homeostasis.

## Introduction

Arachnoid granulations are invaginations of the arachnoid meninges into the dural sinuses and were first described in detail by Pacchioni in the early 18th century, who called them “glandulae congoblatae” (Brunori et al., 1993). He proposed that “glandulae congoblatae” primarily have a secretory function and that fluid created by them “lubricates” the meninges and brain. According to the generally accepted traditional concept of cerebrospinal fluid physiology, arachnoid granulations and arachnoid villi are thought to be the major site of cerebrospinal fluid absorption into the venous system. Although some other possible sites of absorption have been identified in recent years (Gomez et al., 1985; Kida et al., 1993; Brinker et al., 1997; Johnston et al., 2004; Iliff et al., 2013; Rasmussen et al., 2018), arachnoid granulations and villi are still considered a key site of cerebrospinal fluid absorption (Von Monakow, 1905; Weed, 1935; Davson et al., 1973; Milhorat, 1975; Pollay, 2010; Sakka et al., 2011; Damkier et al., 2013; Bothwell et al., 2019). The presumed mechanism of cerebrospinal fluid absorption into the dural sinuses still is not completely clear. It is important to note that there is no scientifically accepted methodology for measuring cerebrospinal fluid absorption. Weed hypothesized that absorption through arachnoid granulations was primarily determined by the difference in hydrostatic and colloidosmotic pressure between the subarachnoid space and the dural venous sinuses (Weed, 1923, 1935). Welch and Friedman (1960) hypothesized the existence of large open channels on arachnoid granulations that act as one-way valves and allow direct drainage of cerebrospinal fluid into dural sinuses due to pressure gradient between cerebrospinal fluid and dural sinuses. However, analysis of arachnoid granulations by electron microscopy did not show the existence of such “valves” (Shabo and Maxwell, 1968a, b). On the contrary, it has been demonstrated that arachnoid granulations and villi are entirely covered by endothelial cells interconnected by “tight junction” connections, which questions the role of arachnoid granulations in cerebrospinal fluid absorption. Still, the traditional concept of cerebrospinal fluid physiology was supported by the observation that transcellular vacuoles in endothelial cells could theoretically be a mechanism of fluid absorption (Tripathi and Tripathi, 1974).

The numerous experimental results cannot fit into the traditional concept of cerebrospinal fluid physiology based on the postulates of secretion, unidirectional circulation, and absorption of cerebrospinal fluid predominantly in dural sinuses. These results suggest that both the secretion and resorption of cerebrospinal fluid take place along the entire capillary network of the central nervous system (Orešković et al., 2003, 2016, 2017c, 2018; Klarica et al., 2009, 2013, 2014, 2019; Bulat and Klarica, 2011; Jurjević et al., 2011; Radoš et al., 2014; Orešković and Klarica, 2014; Orešković et al., 2018). Since the role of arachnoid granulations in the regulation of intracranial fluids is not clear, in this article, we wanted to examine in more detail how the number, size, and distribution of arachnoid granulations change during lifespan from infancy to 80 years of age.

## Materials and Methods

### Subjects

Patients scanned from 2015–2020 were randomly selected from an extensive MRI database at the Neuron Polyclinic at the Croatian Institute for Brain Research. All patients or their legal guardians have given written consent that the MRI scan results can be used for scientific research and education. The medical diagnoses for which the MR examinations were made were as follows: dizziness, syncope, migraine, trigeminal neuralgia, transient ischemic attack, epilepsy, premature birth, concussion, cavernous malformation, deafness, depression, tinnitus. The inclusion criteria for this study were the appropriate age at the time of imaging and age-appropriate findings of CSF system. The exclusion criteria were the existence of pathology that could theoretically disrupt the physiology of cerebrospinal fluid (hydrocephalus, conditions after extensive neurosurgery, conditions after placement of drainage catheters, the presence of expansive processes, developmental malformations, etc.). The age groups of 0 years (neonates), 2 years, and 10 years, 20 years, 40 years, 60 years, and 80 years were analyzed in this study. The patients analyzed as neonates were re-analyzed at 2 years of age, while all other groups were analyzed only once. Re-scanned patients are part of a cohort of 380 preterm infants and only patients with normal MR exams and normal neuropediatric findings are included in the study. Each age group consisted of 10 male and 10 female patients.

### MRI Acquisition

All MRI scans were performed on a 3T MR device (Magnetom PrismaFIT, Siemens, Germany) as part of standard neuroradiological diagnostics using a 64-channel head and neck coil. Coronal T2 sections (TR/ TE = 5,000/100 ms, matrix: 512 × 308; voxel size: 1 × 1 × 3 mm) and axial T2 sections (TR/TE = 5,000/100 ms, matrix: 512 × 282; voxel size: 1 × 1 × 3 mm) were used for morphological quantification of arachnoid granulations. Neonates were scanned under a neuropediatrician’s supervision and with phenobarbitone sedation (5–10 mg/kg). Two-year-old children were imaged under the anesthesiology team’s supervision under general anesthesia using 8% inhaled anesthetic sevoflurane for sedation and 6% for maintenance anesthesia during MR imaging. Subjects in all older age groups were scanned without sedation and anesthesia.

### Image Analysis

**Figure 1:**
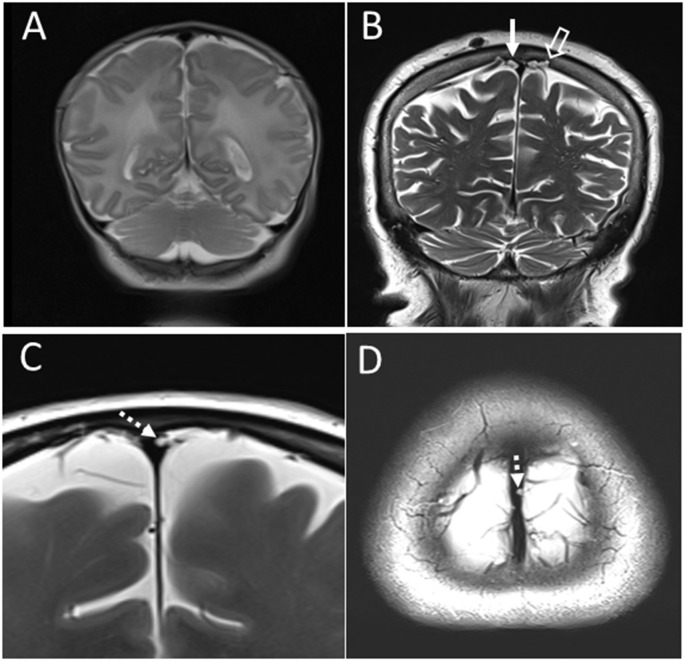
**(A)** Coronal T2 section in neonates without arachnoid granulation in the superior sagittal sinus and the surrounding cranial bones. **(B)** Coronal T2 section in an 80-year-old subject with pronounced arachnoid granulations in the superior sagittal sinus (solid arrow) and the surrounding cranial bones (empty arrow). **(C)** Coronal T2 section in a 60-year-old subject with an example of arachnoid granulation in the superior sagittal sinus has the largest diameter in the coronal plane (dashed arrow). **(D)** Axial T2 section from the same subjects with the representation of the same arachnoid granulation in the superior sagittal sinus (dashed arrow).

MR image analysis was performed using the PACS system (Picture Archiving and Communication System) Carestream (Carestream Health Inc., Rochester, NY, USA) in which arachnoid granulations were analyzed and measured in two planes (coronal and axial). Arachnoid granulation was defined as an impression into the lumen of the venous sinus or into the cranial bone, whose signal on all MRI sequences was the same as the cerebrospinal fluid’s signal. Arachnoid granulations were analyzed in two planes, and the dimension was determined according to the largest diameter, as shown in Figure 1. Arachnoid granulations were classified into seven groups according to size (0–2.0 mm, 2.1–4.0 mm, 4.1–6.0 mm, 6.1–8.0 mm, 8.1–10.0 mm, 10.1–15.0 mm, 15.1–20.0 mm). Arachnoid granulations were measured separately for the dural sinuses (superior sagittal sinus, transverse and sigmoid sinuses) and separately for the surrounding cranial bones.

### Statistical Analysis

The *χ*^2^ test was used to examine the significance of differences in the frequency of arachnoid granulations with respect to age. Fisher’s exact test was used in the case of a significant test for *post hoc* significance testing. A nonparametric test for multiple independent Kruskal-Wallis samples was used to examine differences in arachnoid granulation size concerning age, while a Mann-Whitney U test with Bonferroni correction for multiple testing was used for *post hoc* monitoring of differences. A significance level of *p* < 0.05 or less according to the Bonferroni correction was used. The online calculator GraphPad^1^ was used for the *χ*^2^ test, while all other analyses were performed using SPSS version 21.0.

## Results

### Percentage of the Subjects With Arachnoid Granulations in Dural Sinuses

**Figure 2:**
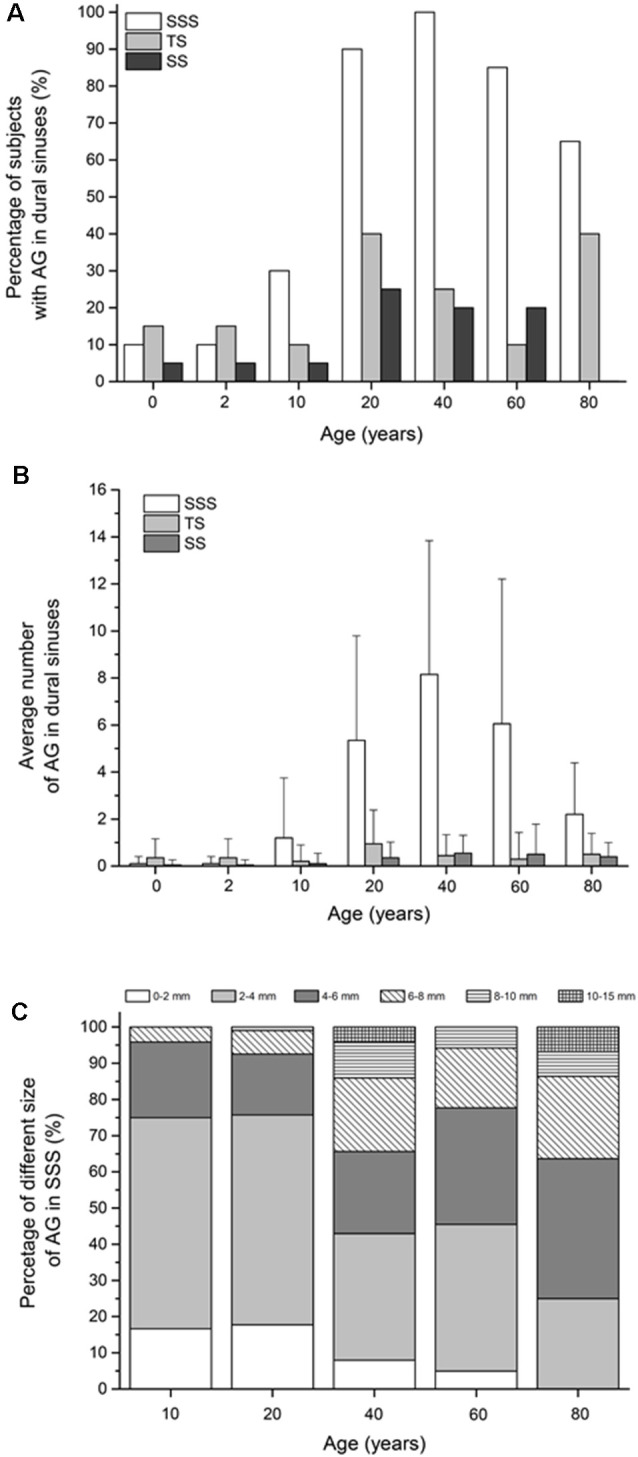
**(A)** Percentage of subjects with arachnoid granulation in dural sinuses. **(B)** Average number of arachnoid granulations in dural sinuses (mean ± standard deviation). **(C)** Proportions of arachnoid granulations of different sizes in the superior sagittal sinus in different age groups. In C only age groups in which the total number of all arachnoid granulations for the whole group was greater than 10 were shown. Abbreviations: SSS, superior sagittal sinus; TS, transverse sinus; SS, sigmoid sinuses.

Our results show that during the lifespan, the proportion of subjects with arachnoid granulations in the dural sinuses changes significantly (Figure 2A). As many as 85% of subjects on MRI sections do not have any visible arachnoid granulation in any of the dural sinuses in the neonatal age. At this age, arachnoid granulations are found in the transverse sinuses in 15% of subjects, in the superior sagittal sinus in 10% of subjects, and in the sigmoid sinuses in 5% of subjects. The same group of children was re-scanned at the age of 2 years, and, interestingly, the results were the same, which shows no change in the number of arachnoid granulations in this period.

The percentage of the subjects with arachnoid granulations in the superior sagittal sinus is significantly different concerning age ($\chi_{\left(6\right)}^{2}$ = 30.40, *p* < 0.0001), with a significantly higher percentage of the subjects with arachnoid granulations at the age of 20 compared to age 10 years (*p* = 0.0002). At the age of 40, arachnoid granulations are present in 100% of subjects, and then a significant trend of a lower percentage of the subjects with arachnoid granulations can be observed at the age of 80 years (*p* = 0.0083) when 65% of subjects have visible arachnoid granulations.

In the transverse and sigmoid sinuses, the share of subjects with arachnoid granulations in all examined age groups never exceeds 40% and 25%, respectively. Regarding arachnoid granulations in transverse sinuses, there are no significant differences in the percentage of the subjects with arachnoid granulations with respect to age ($\chi_{\left(6\right)}^{2}$ = 9.23, *p* = 0.1609). Also, there are no significant differences in the percentage of the subjects with arachnoid granulations in sigmoid sinuses with respect to age ($\chi_{\left(6\right)}^{2}$ = 10.09, *p* = 0.1210).

### The Average Number of Arachnoid Granulations in the Dural Sinuses

Our results show that the average number of arachnoid granulations in the dural sinuses is different at different ages (Figure 2B). Specifically, significant differences with respect to age were found in the average number of granulations in the superior sagittal sinus (*H*_(6)_ = 75.21, *p* < 0.0001), in the transverse (*H*_(6)_ = 14.48, *p* = 0.0247) and sigmoid sinuses (*H*_(6)_ = 16.32, *p* = 0.0121). The largest number of arachnoid granulations is located in the superior sagittal sinus. The average number of arachnoid granulations in the superior sagittal sinus at the ages 0 and 2 years is very low (0.1 ± 0.3). In subjects older than 2 years, the number of arachnoid granulations in the superior sagittal sinus was significantly higher, as shown by the *post hoc* Mann-Whitney U test (*U* = 90.00, *r* = 0.47). We find 1.2 ± 2.5 arachnoid granulations at the age of 10 and 5.4 ± 4.4 and 8.2 ± 5.7 in the age groups of 20 and 40. In older age groups of 60 and 80 years, the average number of arachnoid granulation decreases to 6.1 ± 6.1 and 2.2 ± 2.2, respectively, which is significantly lower (*U* = 55.00, *p* < 0.0001). The average number of arachnoid granulations in the transverse sinuses is highest in the age group of 20 years (1.0 ± 1.4; *H*_(6)_ = 14.48, *p* = 0.0247), while in other age groups, their average number in the transverse sinuses is equal to or less than 0.5. In the sigmoid sinuses, the average number of arachnoid granulations is highest in the age group of 40 years (0.6 ± 0.8; *H*_(6)_ = 16.32, *p* = 0.0121) while in all other age groups, it is equal to or less than 0.4.

### Size of Arachnoid Granulations in Dural Sinuses

In our subjects, arachnoid granulations in the superior sagittal sinus also differed significantly in size, from the smallest measuring less than a millimeter to the largest, measuring 10–15 mm. Figure 2C shows significant difference in the proportions of arachnoid granulations of different sizes in the age groups from 10 to 80 years ($\chi_{\left(20\right)}^{2}$ = 73.69, *p* < 0.0001). According to the data in Figure 2C, it can be seen that the proportion of arachnoid granulations that are less than 4 mm shows a downward trend over 10–80 years, while the proportion of arachnoid granulations that are greater than 4 mm is significantly higher in old age groups. At the age of 10 years, the total proportion of arachnoid granulations that are less than 4 mm make up 75% of all arachnoid granulations in the superior sagittal sinus. At the age of 80, the finding is reversed so that the proportion of arachnoid granulations less than 4 mm is 25%.

### Percentage of the Subjects With Arachnoid Granulations in Cranial Bones

**Figure 3:**
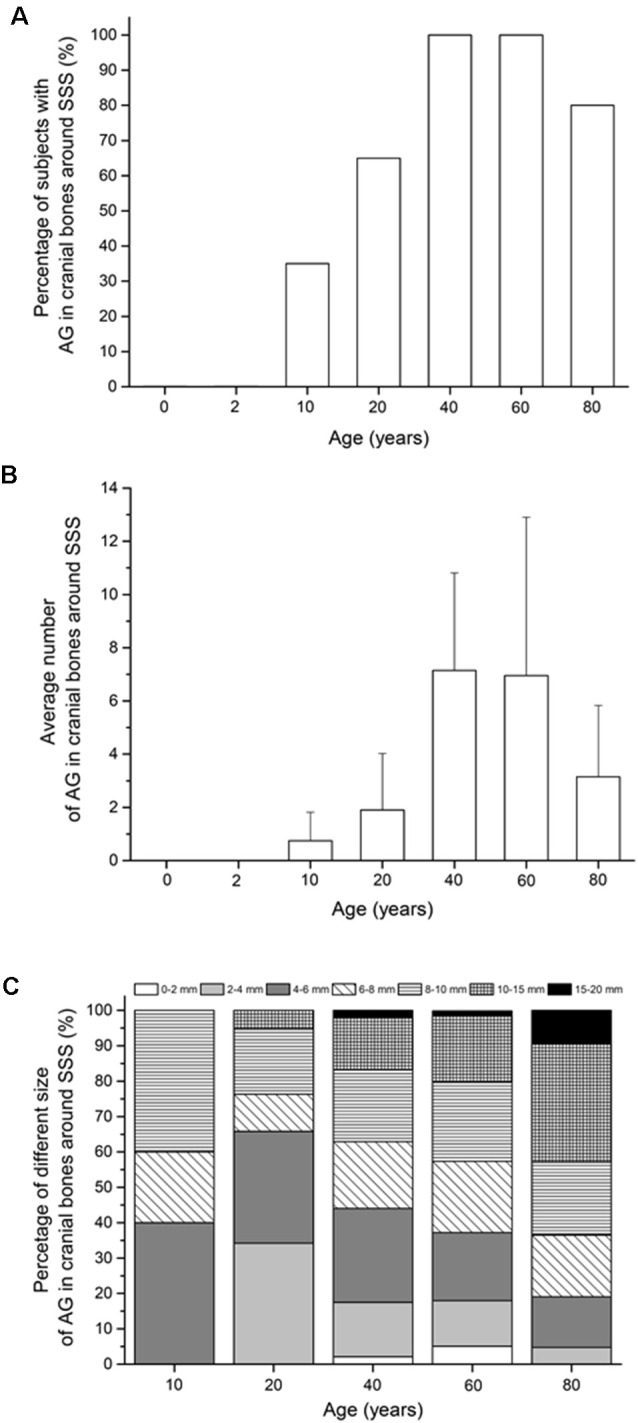
**(A)** Percentage of subjects with arachnoid granulation in the cranial bones around dural sinuses. **(B)** Average number of arachnoid granulations in the cranial bones around superior sagittal sinus (mean ± standard deviation). **(C)** Proportions of arachnoid granulations of different sizes in the cranial bones around superior sagittal sinus in different age groups. In **(C)** only age groups in which the total number of all arachnoid granulations for the whole group was greater than 10 were shown. Abbreviations: SSS, superior sagittal sinus; TS, transverse sinus; SS, sigmoid sinuses.

Arachnoid granulations in the cranial bones were visible only in the bones around the superior sagittal sinus, while in the bones around the transverse and sigmoid sinuses, we did not find arachnoid granulations in any of the subjects (Figure 3A). The percentage of the subjects with arachnoid granulations in the bones around the superior sagittal sinus is significantly different with respect to age ($\chi_{\left(6\right)}^{2}$ = 41.10, *p* < 0.0001). In two groups of the youngest subjects (neonates and subjects aged 2 years), we did not find arachnoid granulations in the bones around the superior sagittal sinus, which indicates that they develop only later in life (*p* < 0.0083). In subjects aged 10 years, arachnoid granulations in the bones around the superior sagittal sinus are found in 35% of subjects, while the percentage of the subjects with arachnoid granulations at age 20 is 65% which is significantly higher (*p* = 0.0083). At age 40 and 60 years, arachnoid granulations are present in 100% of subjects, while in the age group of 80, they are present in 80% of subjects. According to our observation, arachnoid granulations in cranial bones along the superior sagittal sinus are located predominantly in the frontoparietal border area, the most cranial part of the cranium. Most of them are located immediately next to the superior sagittal sinus, but some are located more laterally from the midline (the maximum measurement distance of arachnoid granulation from the midline was 1.5 cm).

### The Average Number of Arachnoid Granulations in Cranial Bones

Furthermore, our results show that the average number of arachnoid granulations in the neurocranial bones (Figure 3B) were significantly different with respect to age only in the bones around the superior sagittal sinus (*H*_(6)_ = 104.06, *p* < 0.0001). The first arachnoid granulations in the cranial bones around the superior sagittal sinus occur in the age group of 10 years (0.6 ± 1.1). In subjects older than 10 years, the average number of arachnoid granulations in the bones around the superior sagittal sinus was significantly higher at the age of 20 years (*U* = 79.00, *p* = 0.0011) and 40 years (*U* = 35.00, *p* < 0.0001) when 1.9 ± 2.1 and at 7.2 ± 3.7 arachnoid granulations were measured. Analysis of the average number of arachnoid granulations in the cranial bones around the superior sagittal sinus shows an increase up to the age of 40. Then the average number of arachnoid granulations decreases (*U* = 55.00, *p* < 0.0001).

### Size of Arachnoid Granulations in Cranial Bones

Arachnoid granulations in the cranial bones around the superior sagittal sinus differed significantly in size, from less than a millimeter to the diameter of 15–20 mm. Figure 3C shows a significant difference in the proportions of arachnoid granulations of different sizes in the age groups from 10 to 80 years ($\chi_{\left(24\right)}^{2}$ = 65.35, *p* < 0.0001). According to the data in Figure 3C, the share of arachnoid granulations of larger diameter is increasing in older age groups. At the age of 10 years, the total proportion of arachnoid granulations that are less than 8 mm make up 60% of all arachnoid granulations in the cranial bones around the superior sagittal sinus. At the age of 80, the finding is reversed so that the proportion of arachnoid granulations less than 8 mm is 40%.

### Comparison of Male and Female Subjects

Comparing results in male and female subjects did not show statistically significant differences in the number, size, and distribution of arachnoid granulations for different age groups.

## Discussion

Our results show that there is a large percentage of subjects in the youngest and oldest age groups who have no arachnoid granulations in the dural sinuses at all (Figure 2A). In the neonates and the 2-year-old children, 85% of the subjects did not have arachnoid granulations in the dural sinuses. Such findings are consistent with previous studies, which showed that before 18 months of age, arachnoid granulation was not visible in most subjects even when the dural sinuses were dissected and observed under a magnifying glass (le Gros Clark, 1920). Furthermore, Figure 2A shows that even in the oldest groups of 60 and 80 years in 15–35% of subjects, there are no arachnoid granulations in the dural sinuses. These findings are also consistent with earlier anatomical studies showing that in adulthood, about 1/3 of subjects have no arachnoid granulations in the dural sinuses (Leach et al., 1996; Grossman and Potts, 1974; Liang et al., 2002). The question arises as to how such findings fit into the classical concept of cerebrospinal fluid physiology, which considers arachnoid granulation to be the most essential cerebrospinal fluid absorption site into the venous system.

It should be noted that arachnoid granulations are considered hypertrophied arachnoid villi. Patients without arachnoid granulations may have much smaller villi which cannot be visualized by MRI (Davson et al., 1973; Papaiconomou et al., 2002). However, histological studies on embryos and fetuses have shown neither arachnoid villi nor arachnoid granulations in the dural sinuses (Osaka et al., 1980; Fox et al., 1996), although choroid plexuses have developed since the third month of gestation (Johanson, 2008). This finding also raises the question of the significance of arachnoid granulations and arachnoid villi in cerebrospinal fluid physiology at the earliest developmental stage. From our results and data from the literature, it seems that arachnoid granulations do not play a key role in absorbing cerebrospinal fluid as assumed by the traditional concept of cerebrospinal fluid physiology because a large number of subjects in all age groups have no arachnoid granulation at all.

The number of arachnoid granulations in the dural sinuses and surrounding bones varies significantly depending on the subjects’ age. The dynamics of changes in the number of arachnoid granulations is most pronounced in the superior sagittal sinus and the cranial bones around it (Figures 2B, 3B). In Figure 2B it can be seen that the average number of arachnoid granulations in the superior sagittal sinus increases from neonatal to 40 years of age. Still, it is interesting that even in the group of 40 years, the number of arachnoid granulations in individual subjects ranges from 1 to 23. All others age groups also have significant interindividual variations in the number of arachnoid granulations. It is to be expected that the small number of arachnoid granulations found in individual subjects in all age groups is not sufficient for a critical role in cerebrospinal fluid absorption as is traditionally assumed. Also, a considerable number of arachnoid granulations have been observed in the bones around the superior sagittal sinus, which certainly do not participate in cerebrospinal fluid absorption. Namely, in the age groups of 60 and 80, arachnoid granulations in the bones around the superior sagittal sinus are more numerous than in the superior sagittal sinus itself (Figures 2B, 3B).

In the transverse and sigmoid sinuses, there is no significant increase in the percentage of the subjects with arachnoid granulations during life, and in the surrounding neurocranial bones, no arachnoid granulations were found in any age group. Although in clinical practice, they can sometimes be seen in bones around transverse and sigmoid sinuses.

Presented results question the general physiological role of arachnoid granulations. It is known that in other organ systems, there are structures that are not present from birth. Their development in later life does not necessarily mean that they have a physiological role but may result from long-lasting pathophysiological processes. One example is the diverticula that develop on the colon’s wall as small protrusions of the mucosa and submucosa through the muscle layer at the site of entry of blood vessels into the wall of the colon (Brian West, 2006). Diverticula that have no physiological role are extremely rare in children but are found in 58% of people over 60 (Peery et al., 2016). Their development is thought to be a consequence of the long-term interaction of pressures within the intestinal lumen and biophysical and genetic characteristics of the colon wall. Interestingly, arachnoid granulations in the dural sinuses are also found predominantly at the site where the drainage veins pass through the wall of the dural sinuses (le Gros Clark, 1920). Therefore, it seems to us that the development of arachnoid granulations could be due to a long-term biophysical interaction of intracranial cerebrospinal fluid pressure and surrounding bone-fibrous and vascular structures. Consequently, it is very possible that arachnoid granulations have no role at all in the physiology of cerebrospinal fluid. When it comes to arachnoid granulations in the cranial bones, it is even more difficult to find any reasonable explanation for their physiological role, even on a theoretical level.

One of the founders of the traditional concept of cerebrospinal fluid physiology, Walter E. Dandy, was very critical of the hypothesis that cerebrospinal fluid is resorbed into the venous system through arachnoid granulations. He considered that cerebrospinal fluid is absorbed on the capillary network of the arachnoid meninges (Dandy, 1929). Studies with protein markers have shown that after their application into the interstitial space or cerebrospinal fluid, these markers can later be found in the extracranial lymphatic system, both in experimental animals and in humans (Bradbury et al., 1981; Löwhagen et al., 1994; Caversaecio et al., 1996). These findings have resulted in the development of the concept of an alternative cerebrospinal fluid drainage pathway into the lymphatic system, predominantly via arachnoid sheaths that penetrate the olfactory nerves through the cribriform plate of the ethmoid bone (Gomez et al., 1985; Kida et al., 1993; Brinker et al., 1997). Furthermore, research on rodents and humans, using molecular markers of different sizes and non-invasive brain imaging techniques, indicates the existence of a glymphatic system within which substances move along arterial perivascular spaces and are ultimately removed through the meningeal or cervical lymphatic system (Johnston et al., 2004; Iliff et al., 2012, 2013; Rasmussen et al., 2018). Despite research indicating alternative cerebrospinal fluid drainage pathways, the prevailing hypothesis is that arachnoid granulations are the dominant site of cerebrospinal fluid absorption.

Presented results fit well with 40-year-long research of our group performed on different species of animals and humans, which show that none of the classical concept settings is accurate. Monitoring the fate of different markers, it seems that there is no active secretion of cerebrospinal fluid predominantly in the ventricles. Consequently, there is no unidirectional circulation of cerebrospinal fluid from ventricles to subarachnoid spaces (Orešković et al., 2002, 2001; Orešković and Klarica, 2010, 2011; Bulat and Klarica, 2011; Orešković et al., 2017a,b,c; Klarica et al., 2019). The distribution of test substances in all directions within the cerebrospinal fluid system, and the neuroradiologically demonstrated uniform oscillatory movement of cerebrospinal fluid volume back and forth under physiological conditions, indicate that there is no unidirectional net movement of cerebrospinal fluid volume (Yamada, 2014; Yamada and Kelly, 2016). According to the Bulat-Orešković-Klarica hypothesis, interstitial fluid is created at the capillary network within the entire central nervous system. It means that each part of the capillary network is both the site of secretion and the site of absorption of interstitial fluid depending on the balance of osmotic forces and hydrostatic pressures in the capillary system and interstitium, which is very clearly shown in the model of isolated brain ventricles (Klarica et al., 2009; Maraković et al., 2010). The interstitial and cerebrospinal fluid spaces are interconnected and could be considered a single functional unit. Thus, according to this concept, cerebrospinal fluid absorption can occur anywhere within the capillary network of neural tissue, and it is entirely independent of arachnoid granulations and arachnoid villi.

## Limitations

There are several limitations to this study that should be mentioned. The first limitation is that all patients came to the MR exam due to a medical indication. Although their diagnoses are not expected to affect CSF physiology, this effect cannot be completely ruled out on a theoretical level.

The second limitation relates to sample size. Although almost all comparisons examined in our study were established to be significantly different, a larger sample size would reduce the possibility of sampling errors.

The third limitation is that the same patients were MR scanned only at the ages of 0 and 2 years when there was no change in the number of arachnoid granulations. It is impossible to say unambiguously from our study whether arachnoid granulations appear or disappear over time in an individual subject. This question could be answered only by conducting a longitudinal study in which the same subjects would be followed for a more extended period, ideally throughout life.

## Conclusion

The results suggest that the number, size, and distribution of arachnoid granulations in the superior sagittal sinus and surrounding cranial bones change significantly over a lifetime. However, numerous individuals do not have or have a minimum number of arachnoid granulations in all age groups. Furthermore, after the age of 60 years, arachnoid granulations are predominantly located in the cranial bones. All of the above suggests that arachnoid granulations do not seem to play a crucial role in cerebrospinal fluid absorption as it is generally accepted. Therefore, the lack of arachnoid granulations does not appear to cause problems in intracranial fluid homeostasis.

## Data Availability Statement

The raw data supporting the conclusions of this article will be made available by the authors, without undue reservation.

## Ethics Statement

The studies involving human participants were reviewed and approved by The ethics committee of Polyclinic Neuron, Šalata 12, 10,000 Zagreb. Written informed consent to participate in this study was provided by the participants’ legal guardian/next of kin.

## Author Contributions

MR, MK, and DO designed and conceptualized the study. MR collected MRI scans. MŽ and AP analyzed MRI scans. MR, MŽ, and AP prepared the figures. MR drafted the initial version of the manuscript. MK and DO provided revisions on the manuscript and explained the presented results according to their new hypothesis of CSF physiology. All authors contributed to the article and approved the submitted version.

## Conflict of Interest

The authors declare that the research was conducted in the absence of any commercial or financial relationships that could be construed as a potential conflict of interest.

## Footnotes

^1^https://www.graphpad.com/
